# SARS-CoV-2 Infection Positively Correlates with Hyperglycemia and Inflammatory Markers in COVID-19 Patients: A Clinical Research Study

**DOI:** 10.3390/diseases12070143

**Published:** 2024-07-04

**Authors:** Prashanth Chikkahonnaiah, Siva Dallavalasa, SubbaRao V. Tulimilli, Muskan Dubey, Shashidhar H. Byrappa, Raghavendra G. Amachawadi, SubbaRao V. Madhunapantula, Ravindra P. Veeranna

**Affiliations:** 1 Department of Pulmonary Medicine, Mysore Medical College and Research Institute, Mysuru 570001, Karnataka, India; prshnthcr@gmail.com; 2 Center of Excellence in Molecular Biology and Regenerative Medicine (CEMR) Laboratory (DST-FIST Supported Centre and ICMR Collaborating Center of Excellence–ICMR-CCoE), Department of Biochemistry (DST-FIST Supported Department), JSS Medical College, JSS Academy of Higher Education & Research (JSS AHER), Mysuru 570015, Karnataka, India; sivakumar65d@gmail.com (S.D.); subbaraotulimilli23@gmail.com (S.V.T.); 3 Xavier University School of Medicine, Xavier University School of Veterinary Medicine, Santa Helenastraat #23, Oranjestad, Aruba; muskan.dubey@students.xusom.com; 4 Department of Pathology, Mysore Medical College and Research Institute (MMC&RI), Mysuru 570001, Karnataka, India; shbmysore@gmail.com; 5 Department of Clinical Sciences, College of Veterinary Medicine, Kansas State University, Manhattan, KS 66506, USA; agraghav@vet.ksu.edu; 6 Leader, Special Interest Group in Cancer Biology and Cancer Stem Cells (SIG-CBCSC), JSS Medical College, JSS Academy of Higher Education & Research (JSS AHER), Mysuru 570004, Karnataka, India

**Keywords:** SARS-CoV-2, COVID-19, hyperglycemia, hematological variables, inflammatory parameters

## Abstract

Diabetes mellitus (DM) is a common comorbidity in COVID-19 subjects. Hyperglycemia at hospital admission identified as a major risk factor and is responsible for poor prognosis. Hematological and inflammatory parameters have been recognized as predictive markers of severity in COVID-19. In this clinical study, we aimed to assess the impact of hyperglycemia at hospital admission on hematological and several inflammatory parameters in COVID-19 patients. A total of 550 COVID-19 subjects were primarily categorized into two major groups (normoglycemic and hyperglycemic) based on random blood sugar levels. On the first day of hospitalization, subjects’ oxygen saturation, random blood sugar, hematological variables, and inflammatory parameters were recorded. The hyperglycemic group exhibited higher levels of serum ferritin, total leukocyte count (TLC), lactate dehydrogenase (LDH), neutrophil count, and neutrophil-to-lymphocyte ratio (NLR). In contrast, oxygen saturation and lymphocyte count were lower compared to the normoglycemic group. Significantly elevated levels of hematological variables (TLC, neutrophil count, NLR) and inflammatory parameters (serum ferritin) were observed in the hyperglycemic group. Among inflammatory parameters, only serum ferritin levels showed statistical significance. This study supports the clinical association between hyperglycemia and an increased severity of COVID-19. Consequently, the identification of these parameters is a crucial and valuable prognostic indicator for assessing disease severity in hyperglycemic subjects.

## 1. Introduction

The COVID-19 pandemic was triggered mainly by severe acute respiratory syndrome coronavirus-2 (SARS-CoV-2), structurally a single-stranded, positive-sense, enveloped RNA-associated virus capable of infecting both humans and animals [1]. SARS-CoV-2 mainly enters the target cells with the help of angiotensin-converting enzyme (ACE)-2 receptors, utilizing the endosomal pathway. Following cell entry, the virus undergoes replication, leading to the apoptosis of the target cell and the release of mature virions. These virions subsequently infect neighboring cells, activating pro-inflammatory pathways that result in a cytokine storm, eventually leading to tissue fibrosis and loss of function in the affected organ. Numerous study reports have detailed that the severity of COVID-19 infection is heightened in individuals with several underlying comorbidities, like diabetes, obesity, hypertension, and cardiovascular diseases, among others [2]. Earlier studies from the Chinese Centre for Disease Control and Prevention have reported 72,314 new cases and revealed an increased mortality rate in individuals with diabetes (7.3% for diabetic subjects compared to 2.3% for non-diabetic subjects) [3]. In Hong Kong, among the total COVID-19-positive cases, 42.3% were diabetic subjects, with 26 fatalities reported in diabetic subjects aged 75 years and above [4]. A recent study conducted by Guan et al. (2020) demonstrated that of a total of 1099 subjects with COVID-19, 16.2% reported diabetes mellitus, 23.7% had hypertension, 5.8% reported coronary heart diseases, and 2.3% had cerebrovascular disease, indicating severity in cases with comorbidities [5]. In another study involving 140 COVID-19 subjects, 12% had diabetes, and 30% had hypertension [6].

In a multivariate analysis, it was found that diabetes independently correlates with increased mortality in individuals with COVID-19 infection [7]. The presence of insulin resistance and hyperglycemic conditions in diabetes stimulates pro-inflammatory cytokines synthesis, the advanced glycation process of end products (AGEs), and oxidative stress. Additionally, it mainly triggers the production of adhesion molecules, mediating inflammation in the tissue levels [8,9]. This pro-inflammatory response appears to be a potential underlying mechanism for the heightened infections and severity observed in subjects with diabetes, leading to worse outcomes.

Conversely, studies suggest that the SARS-CoV-2 infection alone can elevate blood glucose levels in individuals without a history of hyperglycemia. Subjects with COVID-19 have been observed to experience new-onset diabetic symptoms and severe metabolic complications, such as ketoacidosis and hyperosmolarity, etc. [10,11,12]. European pediatric populations reported an increase in type 1 diabetes among individuals under 18 years of age within 30 days of infection, while in adults it may manifest as a long-term consequence of the viral infection [13]. The virus infects cells through ACE-2 receptors, which are mainly expressed in key metabolic-associated organs, tissues like pancreatic beta cells, adipose tissue, the small intestine, and the kidney [14]. This suggests that SARS-CoV-2 may induce diverse changes in blood glucose levels, with glucose metabolism complicating the pathophysiology of preexisting diabetes or leading to new-onset diabetes. Furthermore, it has been observed that SARS-CoV-2 infection results in the release of high levels of glucocorticoids and catecholamines, further elevating blood glucose [15]. These findings highlight the bidirectional relationship between COVID-19 and diabetes. Several studies have previously reported that hematological and inflammatory parameters serve as predictive markers of severity in COVID-19 subjects, and assessing these parameters can aid in planning the management of COVID-19 subjects [16,17,18,19]. In this study, our goal was to investigate the impact of hyperglycemia at hospital admission on the hematological and inflammatory parameters in COVID-19 subjects.

## 2. Materials and Methods

### 2.1. Study Design, Ethical Considerations, Data Collection, and Case Enrollment

An observational cross-sectional cohort study was mainly conducted among subjects diagnosed with COVID-19, identified either through a rapid antigen test or RT-PCR; subjects were admitted to a designated COVID hospital in Mysuru, Karnataka, India. Preparatory approvals were taken from the institutional ethics committee (MMCEC36/2021). Written consent, RT-PCR test reports, and random blood sugar (RBS) levels were considered for patient enrollment. Subsequently, subjects were mainly categorized into two groups based on their random blood sugar levels: normoglycemic (RBS < 140 mg/dL) and hyperglycemic (RBS > 140 mg/dL).

The study involved the examination of patient blood for various parameters, including serum ferritin, lactate dehydrogenase, random blood sugar levels, total leukocyte count, neutrophil count, lymphocyte count, and neutrophil-to-lymphocyte ratio. The research was conducted at the Mysore Medical College and Research Institute (MMC&RI) (Department of Respiratory Medicine) and Princess Krishnajammanni Trauma Care Center (PKTCC) (which served as a designated COVID hospital), Mysore; both male and female participants were included in the present study. All the samples were processed at the central laboratory facility, Krishna Rajendra Hospital, Mysore, Karnataka, India.

### 2.2. Sample Collection and Processing

On the day of admission, after obtaining informed consent, about 5.0 mL of venous blood was collected from each subject aseptically and divided into two fractions, one for hematological (purple color-coded, EDTA anticoagulant tubes) and the other for serological (red color-coded, no anticoagulant tubes) examination. The blood collected for hematological examination was used for estimating random blood sugar levels, total leukocyte count, neutrophil count, lymphocyte count, and neutrophil-to-lymphocyte ratio. The blood collected for serological examination was allowed to clot by leaving it undisturbed at room temperature for 30 min. After coagulation, the serum was separated by centrifugation at 3000 rpm for 10 min at room temperature. Serum ferritin (determined using the chemiluminescence method, as detailed below) and serum LDH (estimated using a kit-based method-LDHI2 reagent kit Cat. No. 03004732122) were measured using a Cobas 6000 Chemistry Analyzer from Roche Diagnostics, Indianapolis, IN, USA, which were available at the central laboratory facility, Krishna Rajendra Hospital, Mysore, Karnataka, India.

### 2.3. Estimation of Serum Ferritin

Serum ferritin levels were determined using the chemiluminescence method (COBAS 6000 from Roche Diagnostics, Indianapolis, IN, USA). The assay works on a sandwich principle, where a ferritin-defined antibody and a specific labeled ferritin antibody form a sandwich complex in the primary step. In the second step, the specific microparticles were introduced to facilitate the sandwich for binding to a solid phase. Further, the reaction mixture was aspirated into the measuring cells and captured microparticles onto the electrode. After removing the unbound microparticles, a specific voltage was applied, inducing chemiluminescence; the emitted chemiluminescence was captured and measured by a photomultiplier, and readings were noted and used to quantify the serum ferritin through a calibration curve.

### 2.4. Statistical Analysis

The study findings encompass the both descriptive and inferential statistical analyses which were carried out. The results for continuous variables were presented as Mean ± SEM, while the results for categorical variables were expressed as frequency (percentage). A statistical analysis was performed using GraphPad Prism 5, and a *p*-value of <0.05 was considered statistically significant.

## 3. Results

### 3.1. Clinical Characteristics

Among the 550 subjects included in the study, 354 (64.36%) were males. The majority, comprising 368 (66.90%) of the subjects, fell within the age group of 30–60 years. A significant proportion, 442 (80.36%) of the subjects, had not received vaccination. More than 68% of the total subjects reported experiencing fever and cough, while 45% experienced breathlessness. In the normoglycemic group (202 subjects), 127 (62.87%) were males, and 136 (67.32%) were in the age group of 30–60 years. The majority, 159 (78.71%), were unvaccinated. More than 64% of these subjects reported symptoms of fever and cough, and 41% experienced breathlessness. Thirty-two of 202 (32/202 = 15.84%) normoglycemic participants had preexisting diabetes.

In the hyperglycemic group (*n* = 348), 227 (65.22%) were males. The majority (*n* = 232; 66.66%) of hyperglycemic participants were in the age group of 30–60. The number of unvaccinated participants was found to be very high in the hyperglycemic group (*n* = 283; 81.32%). More than 70% of these subjects reported symptoms of fever and cough, while 47% experienced breathlessness. Interestingly, whereas 225 (64.65%) of 348 hyperglycemic subjects developed new-onset hyperglycemia, 126 (36.20%) had preexisting diabetes (Table 1).

### 3.2. Inflammatory Parameters

**Figure 1:**
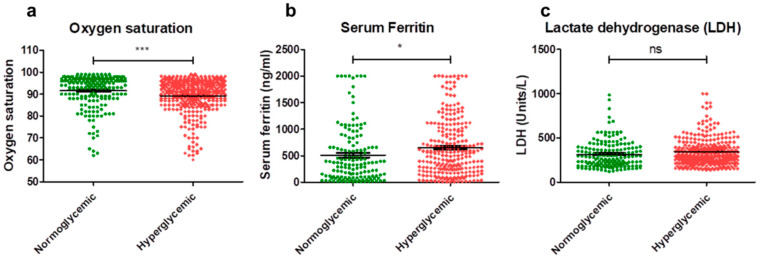
Inflammatory parameters in normoglycemic and hyperglycemic subjects infected with SARS-CoV-2. The dot plots illustrate the mean (Mean ± SEM) oxygen saturation levels (**a**), serum ferritin (**b**), and LDH levels (**c**) in admitted subjects, comparing normoglycemic and hyperglycemic groups. Statistical comparison between the two groups was conducted using an unpaired *t*-test (two-tailed), and a *p*-value < 0.05 was considered statistically significant. * *p* < 0.05 and *** *p* < 0.001, NS: Non-Significant.

In this current study, the mean oxygen saturation levels were 91.55% in normoglycemic COVID-19-infected subjects and 89.24% in hyperglycemic COVID-19-infected subjects. An analysis of the data revealed a significant difference, with normoglycemic subjects exhibiting a higher mean oxygen saturation of 91.55%, compared to hyperglycemic subjects, with a mean saturation of 89.24% (Figure 1a).

The mean serum ferritin levels were 509.5 ng/mL in normoglycemic subjects and 650.0 ng/mL in hyperglycemic subjects. An analysis of the data revealed a significant difference, with hyperglycemic subjects exhibiting a higher mean serum ferritin level of 650.0 ng/mL compared to normoglycemic subjects, who had a mean level of 509.5 ng/mL (Figure 1b).

The mean LDH levels were 315.9 units/L in normoglycemic COVID-19-infected subjects and 344.3 units/L in hyperglycemic subjects. An analysis of the data indicated that there was no significant difference observed between the mean LDH levels of normoglycemic and hyperglycemic COVID-19-infected subjects (Figure 1c).

### 3.3. Hematological Parameters

**Figure 2:**
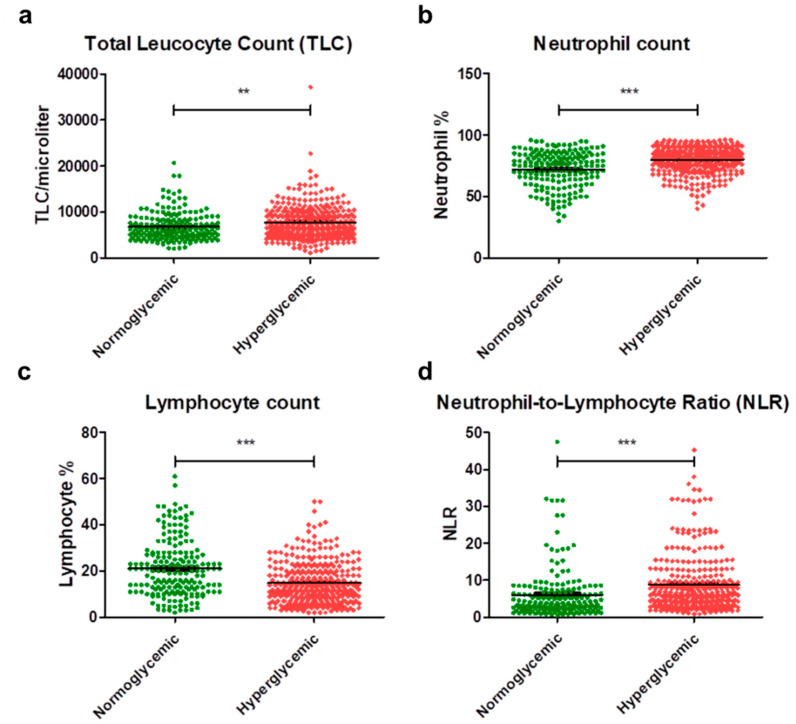
Hematological parameters in normoglycemic and hyperglycemic subjects infected with SARS-CoV-2. The dot plots illustrate the mean (Mean ± SEM) total leukocyte count (**a**), neutrophil count (**b**), lymphocyte count (**c**), and neutrophil to lymphocyte ratio (**d**) in admitted subjects, comparing normoglycemic and hyperglycemic groups. Statistical comparison between the two groups was conducted using an unpaired *t*-test (two-tailed), and a *p*-value < 0.05 was considered statistically significant. ** *p* < 0.01 and *** *p* < 0.001.

In this study, the mean TLC was 6814/microliter in normoglycemic COVID-19-infected subjects and 7699/microliter in hyperglycemic subjects. An analysis of the data revealed a significant difference, with hyperglycemic subjects showing a higher mean TLC of 7699/microliter compared to normoglycemic subjects with a mean TLC of 6814/microliter (Figure 2a).

The mean neutrophil count was 72.16% in normoglycemic COVID-19-infected subjects and 80.10% in hyperglycemic subjects. An analysis of the data revealed a significant difference, with hyperglycemic subjects demonstrating a higher mean neutrophil count of 80.10% compared to normoglycemic subjects with a mean count of 72.16% (Figure 2b).

In the present study, the mean lymphocyte count was 21.05% in normoglycemic COVID-19-infected subjects and 14.77% in hyperglycemic subjects. An analysis of the data revealed a significant difference, with hyperglycemic subjects demonstrating a lower mean lymphocyte count of 14.77% compared to normoglycemic subjects with a mean count of 21.05% (Figure 2c).

The mean neutrophil to lymphocyte ratio (NLR) was 6.034 in normoglycemic COVID-19-infected subjects and 8.769 in hyperglycemic subjects. An analysis of the data revealed a significant difference, with hyperglycemic subjects showing a higher mean NLR of 8.769 compared to normoglycemic subjects with a mean NLR of 6.034 (Figure 2d).

## 4. Discussion

Severe outcomes with higher morbidity and mortality rates in SARS-CoV-2 infected subjects have been associated with various factors, including age and comorbidities such as diabetes, hypertension, obesity, cardiovascular diseases, chronic kidney and liver diseases, low lymphocyte and albumin counts, elevated levels of lactate dehydrogenase, C-reactive protein, red blood cell distribution width, blood urea nitrogen, and direct bilirubin [20,21,22]. Neutrophilia, thrombocytopenia, higher lactate dehydrogenase, and D-dimer levels are more likely to develop Acute Respiratory Distress Syndrome (ARDS), particularly in older subjects [23,24].

Diabetes is one of the more frequent comorbidities of SARS-CoV-2 infection [4,25,26,27], and there is a bidirectional association between SARS-CoV-2 infection and diabetes. On one hand, pre-existing diabetes is associated with an increased risk of severe SARS-CoV-2 infection. On the other hand, severe SARS-CoV-2 infected subjects are prone to new-onset diabetes and severe metabolic complications of pre-existing diabetes, possibly due to adverse immune reactions and the destruction of pancreatic β-cells [10,11,12]. This bidirectional relationship between SARS-CoV-2 infection and diabetes poses challenges in clinical management and suggests a complex pathophysiology of COVID-19 disease.

SARS-CoV-2 binds to angiotensin-converting enzyme 2 (ACE2) receptors, expressed on the cells of key metabolic organs and tissues, including pancreatic beta cells, adipose tissue, small intestine, and kidneys [14]. Thus, SARS-CoV-2 may cause pleiotropic alterations in glucose metabolism that could complicate the pathophysiology of pre-existing diabetes or lead to new mechanisms of disease. Glycemic characteristics predict clinical outcomes, with higher blood glucose levels associated with more extended hospital stays and higher mortality rates in COVID-19 subjects [28]. Improved blood glucose monitoring and management have been shown to result in better outcomes and reduced mortality rates in subjects with SARS-CoV-2 infection [29,30].

Preliminary studies reported an increased prevalence of hyperglycemia in subjects affected by COVID-19 [31,32]. Similar results were reported previously in subjects affected by SARS-CoV-1, which increased fasting blood glucose levels compared to levels observed in subjects with non-SARS-CoV-2 infection pneumonia [33,34,35]. Severe hyperglycemia is common in critically ill subjects and is often considered a marker of disease severity [36]. Several studies over the course of the pandemic have reported that SARS-CoV-2 infection is associated with hyperglycemia in people with or without a history of diabetes [28,37].

In the present study, out of 550 COVID-19 subjects, 348 (63.27%) were found to have random blood sugar levels > 140 mg/dL (considered hyperglycemic), while 202 (36.72%) subjects had levels < 140 mg/dL (considered normoglycemic). The study demonstrated that 15% of the normoglycemic subjects and 36% of the hyperglycemic subjects had preexisting diabetes. Interestingly, 64% of the hyperglycemic subjects had new-onset hyperglycemia, indicating the effect of SARS-CoV-2 infection on the glycemic index. The study also observed, in COVID-19 subjects, a significant association of hyperglycemia with mean oxygen saturation, serum ferritin, total leukocyte count, neutrophil count, lymphocyte count, and neutrophil-to-lymphocyte ratio.

Several people infected with COVID-19 have low oxygen saturation levels, and maintaining oxygen saturation within the target range is crucial for patient management. In the present study, the mean oxygen saturation levels in the hyperglycemic subjects’ group (89.24%) were significantly lower compared to the normoglycemic subjects’ group (91.55%). Prior studies have reported that low blood oxygen saturation is associated with increased mortality, and individuals with diabetes have low blood oxygen saturation compared with non-diabetic controls [38,39]. Another study reported that hyperglycemia-induced cellular hypoxia and mtROS may promote hyperglycemic damage in a coordinated manner [40]. A recent study has shown an elevated ACE2 expression, and its glycated product, in COVID-19 patients who had hyperglycemia. This study has also reported that COVID-19 patients with sustained hyperglycemia had low oxygen saturation levels [41]. In summary, hyperglycemia is one of the critical factors contributing to the low oxygen saturation levels observed in diabetic individuals, and is responsible for various exacerbations reported in diabetic individuals suffering from COVID-19.

Serum ferritin, being an acute-phase reactant, reflects the degree of both chronic and acute inflammatory reactions in the body. High serum ferritin levels are associated with increased disease severity and a poor outcome in people infected with SARS-CoV-2 [42]. In the present study, mean serum ferritin levels in the hyperglycemic subjects’ group (650.0 ng/mL) were significantly higher compared to the normoglycemic subjects’ group (509.5 ng/mL). Although the exact mechanism is not known, a study reported that patients with type 2 diabetes had considerably higher serum ferritin levels, and there was a positive link found between serum ferritin and HbA1c [43].

Serum lactate dehydrogenase (LDH) catalyzes the conversion of pyruvate to lactate, the final step of aerobic glycolysis [44]. An elevated LDH indicates tissue hypoperfusion, which further indicates the extent of the disease [45,46] and is a potential prognostic biomarker in subjects with COVID-19 [47]. However, in the present study, no significant difference was observed between the mean LDH levels of normoglycemic and hyperglycemic COVID-19-infected subjects.

SARS-CoV-2 infection begins as an inflammatory disorder that progresses to severe pneumonia, cytokine storm, acute respiratory distress syndrome, and multi-organ failure, resulting in prolonged hospitalization with poor overall outcomes and potentially death [48]. Several studies have reported that hematological parameters are the predictive markers of severity in COVID-19 subjects [49,50,51]. Analyzing hematological parameters immediately after hospital admission may serve as an effective tool in the early identification of subjects at risk of developing severe disease [52]. Hematological parameters like total leukocyte count, neutrophil count, lymphocyte count, and neutrophil-to-lymphocyte ratio are known to predict the severity of SARS-CoV-2 infection in COVID-19 subjects [50,53,54,55]. These parameters are simple, readily available, and cost-effective prognostic indicators that can be utilized for diagnosis and predicting the severity of COVID-19 infection, particularly in developing countries [56,57,58]. In the present study, the mean total leukocyte count, neutrophil count, and neutrophil-to-lymphocyte ratio were significantly higher, and the lymphocyte count was significantly lower in the hyperglycemic subjects’ group compared to the normoglycemic subjects’ group. Hyperglycemia, along with elevated stress hormones such as cortisol and catecholamines, as well as cytokines, can cause bone marrow progenitor cell expansion, thereby promoting leukocytosis or increased leukocyte count [59]. Although the exact mechanism of the effects of hyperglycemia on lymphocytes is unclear, prior studies have reported that COVID-19 patients with diabetes mellitus do experience a faster decline in lymphocyte count, a lower lymphocyte number, and a longer hospital stay than non-diabetic patients [60,61].

According to a recent cohort study, the long-term persistence of COVID-19 induces hyperglycemia in patients without a previous history of diabetes [62]. The study identified that 77% of the participants continued to receive antidiabetic therapy (oral hypoglycemic agents or insulin) or had persistent hyperglycemia at the end of 6 months [62]. Hyperglycemia, with or without pre-existing diabetes, increases poor outcomes and mortality by aggravating the immune dysfunction (decreased lymphocytes, CD16^+^, 56^+^, NK cells, CD3^+^ T cells, CD8^+^ T cells, and increased neutrophil percentage, and serum concentration of IgA) [63], cytokine storm, elevating pro-inflammatory markers (C-reactive protein, procalcitonin) [64], and inflammatory cytokine production (interleukin 6, tumor necrosis factor-α) [65], as well as coagulation activation (as monitored by plasma D-dimer levels) [66], impaired beta cell function, worsened endothelial dysfunction, etc. [66,67,68].

Stress levels are generally high in critically ill, hospitalized, and COVID-19 patients [69]. Stress-induced hyperglycemia is a common observation seen in 50% of critically ill patients admitted to the hospital [70]. Counter-regulatory hormones (cortisol, catecholamines, glucagon, and growth hormone) disturb glucose hemostasis and pro-inflammatory cytokines (interleukin-1 (IL-1), interleukin-6 (IL-6), and tumor necrosis factor-α (TNF-α)) and worsen the metabolic milieu by causing insulin resistance in stress-induced hyperglycemia [70,71,72]. Glycosylated hemoglobin (HbA1c), which is used to determine an individual’s average blood sugar levels over the previous 2–3 months, is the most widely used monitoring and diagnosing biomarker for diabetes [73]. Along with the inflammatory and hematological parameters reported in the present study, future studies should focus on measuring the glycosylated hemoglobin (HbA1c), insulin, and glucocorticoids in the serum as markers to identify the diabetic individuals at higher risk and provide timely intervention as a priority to improve patient outcomes.

## 5. Conclusions

In conclusion, our study suggests that compared to normoglycemic subjects, hyperglycemic COVID-19-infected subjects showed an increase in lactate dehydrogenase, serum ferritin, total leukocyte count, neutrophil count, and neutrophil-to-lymphocyte ratio, and a decrease in oxygen saturation and lymphocyte count. These results indicate an association between an increased severity of SARS-CoV-2 infection and elevated random blood sugar levels. Whether elevated blood sugar levels in non-diabetic subjects are due to immune-mediated or inflammatory responses, the direct effects of SARS-CoV-2 on β-cells, or a complex combination of mechanisms, is not known. Further studies with long-term follow-up in large cohorts are warranted to confirm the potential association between SARS-CoV-2 infection and an increased risk of diabetes.

## Figures and Tables

**Table 1:** Clinical characteristics of subjects infected with SARS-CoV-2 infection (normoglycemic vs. hyperglycemic).

Clinical Characteristics	Total	Normoglycemic (RBS < 140 mg/dL)	Hyperglycemic (RBS > 140 mg/dL)
Total number of subjects	*n* = 550	*n* = 202	*n* = 348
Gender			
Male	354 (64.36)	127 (62.87)	227 (65.22)
Female	196 (35.63)	75 (37.12)	121 (34.77)
Age distribution			
<30	57 (10.36)	28 (13.86)	29 (8.33)
30–60	368 (66.90)	136 (67.32)	232 (66.66)
>60	125 (22.72)	38 (18.81)	87 (35.08)
Vaccination status			
Unvaccinated	442 (80.36)	159 (78.71)	283 (81.32)
Vaccinated	108 (19.63)	43 (21.28)	65 (18.67)
Single Dose vaccination	75 (69.44)	31 (72.09)	44 (67.69)
Double Dose vaccination	33 (30.55)	12 (27.90)	21 (32.30)
Clinical features			
Fever	379 (68.90)	131 (64.85)	248 (71.26)
Cough	417 (75.81)	149 (73.76)	268 (77.01)
Breathlessness	249 (45.27)	83 (41.08)	166 (47.70)
Preexisting diabetes	158 (28.72)	32 (15.84)	126 (36.20)
New onset hyperglycemia	225 (40.90)	None	225 (64.65)

Data are presented as *n* (%).

## Data Availability

Data are available upon reasonable request. The dataset used to conduct the analyses is available from the corresponding author upon reasonable request.
